# Xenon Treatment Protects Against Cold Ischemia Associated Delayed Graft Function and Prolongs Graft Survival in Rats

**DOI:** 10.1111/ajt.12293

**Published:** 2013-05-24

**Authors:** H Zhao, H R Watts, M Chong, H Huang, C Tralau-Stewart, P H Maxwell, M Maze, A J T George, D Ma

**Affiliations:** 1 Department of Surgery and Cancer, Section of Anaesthetics, Pain Medicine and Intensive Care, Faculty of Medicine, Imperial College London, Chelsea & Westminster HospitalLondon, UK; 2 Department of Medicine, Drug Discovery Centre, Imperial College LondonLondon, UK; 3 Division of Medicine, University College LondonLondon, UK; 4 Department of Anesthesia and Perioperative Care, University of CaliforniaSan Francisco, CA; 5 Section of Molecular ImmunologyDepartment of MedicineImperial College London, Hammersmith HospitalLondon, UK; 6 Department of Anesthesiology, Hubei University of MedicineHubei, China

**Keywords:** Delayed graft function (DGF), graft survival, renal graft loss, treatment

## Abstract

Prolonged hypothermic storage causes ischemia-reperfusion injury (IRI) in the renal graft, which is considered to contribute to the occurrence of the delayed graft function (DGF) and chronic graft failure. Strategies are required to protect the graft and to prolong renal graft survival. We demonstrated that xenon exposure to human proximal tubular cells (HK-2) led to activation of range of protective proteins. Xenon treatment prior to or after hypothermia–hypoxia challenge stabilized the HK-2 cellular structure, diminished cytoplasmic translocation of high-mobility group box (HMGB) 1 and suppressed NF-κB activation. In the syngeneic Lewis-to-Lewis rat model of kidney transplantation, xenon exposure to donors before graft retrieval or to recipients after engraftment decreased caspase-3 expression, localized HMGB-1 within nuclei and prevented TLR-4/NF-κB activation in tubular cells; serum pro-inflammatory cytokines IL-1β, IL-6 and TNF-α were reduced and renal function was preserved. Xenon treatment of graft donors or of recipients prolonged renal graft survival following IRI in both Lewis-to-Lewis isografts and Fischer-to-Lewis allografts. Xenon induced cell survival or graft functional recovery was abolished by HIF-1α siRNA. Our data suggest that xenon treatment attenuates DGF and enhances graft survival. This approach could be translated into clinical practice leading to a considerable improvement in long-term graft survival.

## Introduction

As a mainstream therapeutic method, kidney transplantation has drastically transformed the health quality of patients suffering from end-stage renal diseases. However, improvement of renal graft life-span is far from satisfactory despite of advances in surgical techniques and more powerful immunosuppressants 1, with severe functional impairment occurring in the majority of recipients by 10 years posttransplantation 2. Hypothermic preservation is routinely used to enable long-distance transport and to improve the histocompatibility match of the grafts; however, accumulating laboratory and clinical evidence highlights the importance of ischemia-reperfusion injury (IRI) as a contributing factor in the early delayed graft function (DGF) and the later graft loss 3–4. During prolonged cold ischemia, the absence of blood perfusion to the graft results in severe metabolic imbalance, which promotes tissue necrosis. Reinstitution of blood flow and concomitant reoxygenation is frequently associated with an exacerbation of tissue injury 5. The consequences of IRI are not only the initial ablation of functioning nephrons, but also the activation of the immune response 4–6. This immunity against the post-ischemic graft involves signaling events through damage associated molecular pattern molecules (DAMPs) such as HMGB-1, their binding to toll-like receptors (TLRs), subsequent initiation of NF-κB downstream signaling cascades and the induction of robust inflammatory cytokine production 7, consequently leading to an increased alloresponse and graft injury.

Xenon is used as anesthetic gas due to its narcotic effects and remarkable safety profile 8. Its organoprotective effects on vital organs have been documented in the literature 9–13; in particular, xenon administered prior to warm ischemia-reperfusion injury attenuated kidney injury and also prolonged survival in mice 14. The current work further extends this finding to explore whether xenon, administered to either the donor before grafting or the recipient following grafting, ameliorates early graft injury associated with cold ischemia in a rat model of kidney transplantation. The aims of this study were to evaluate the efficacy of xenon administration in preventing cold ischemia reperfusion injury and to explore the underlying mechanisms of its action both *in vitro* and *in vivo*. Long-term graft outcome through preserving graft quality by xenon was also investigated in the Lewis-to-Lewis and Fischer-to-Lewis kidney transplant models.

## Materials and Methods

### *In vitro* cell culture and hypothermia-hypoxia challenge

Human kidney-2 (HK-2) cells (European Cell Culture Collection) were cultured at 37°C in RPMI 1640 medium and then incubated at 4°C for 24 h in Soltran preserving solution (Baxter Healthcare, Berkshire, UK) in a closed and purpose-built airtight chamber containing 8% O_2_ and 5% CO_2_ balanced with N_2_. Cells were then recovered for 24 h at 37°C in RPMI 1640 medium in normal cell incubator.

### Xenon exposure *in vitro*

Xenon treatment was given either 24 h prior to (pretreatment) or after hypoxia (posttreatment). Cells were incubated with the RPMI medium pre-bubbled with gas mixtures of 70% xenon and 5% carbon dioxide balanced with oxygen, and then exposed to the same gas concentrations for 2 h at 37°C in the gas chamber mentioned above. The treated cells were recovered in RPMI medium in normal incubator at 37°C for 24 h.

### Cell viability *in vitro*

The viability of cells was assessed by using a colorimetric MTT assay (Merck KGaA, Darmstadt, Germany).

### *In vitro* siRNA Transfection

*In vitro* Hypoxia inducible factor-1α (HIF-1α) siRNA transfections were carried out using lipofectamine (Invitrogen, Paisley, UK). siRNA targeting Human HIF-1α (Qiagen, Crawley, West Sussex, UK, Sense strand; 5′-GAAGAACUAUGAACAUAAATT-3′ and antisense strand: 5′-UUUAUGUUCAUAGUUCUUCCT-3′) were dissolved in siRNA suspension buffer and administered to HK-2 cells in a dose of 20 nM, scrambled siRNA served as negative control. Cells were incubated with siRNA for 6 h at 37°C in humidified air containing 5% carbon dioxide, after which it was removed and replaced with experimental medium followed by xenon gas treatment.

### Animals

Inbred adult male Lewis rats and Fischer Rats weighing 225–250 g were purchased from Harlan, UK and bred in temperature- and humidity-controlled cages in a specific pathogen-free facility at Chelsea-Westminster Campus, Imperial College London. This study was approved by the Home Office, United Kingdom, and all animal procedures were carried out in accordance with the United Kingdom Animals (Scientific Procedures) Act of 1986.

### Renal transplantation

Lewis rat (LEW, RT1^1^) to Lewis (LEW, RT1^1^) rat and Fisher (F344, RT1^1vr^) to Lewis rat (LEW, RT1^1^) rat renal transplantations were used as the isograft and allograft model, respectively. Rat donor kidneys were transplanted orthotopically into recipients using standard microvascular techniques. Briefly, the donor left kidney, aorta and inferior vena cava were carefully exposed and the kidney graft was then extracted, flushed and stored in 4°C heparinized Soltran Preserving Solution (Baxter Healthcare). After the specified period of cold ischemia, the recipient's left kidney was extracted and the donor renal vein was connected to the recipient renal vein through end-to-end anastomosis. The arterial anastomosis between the donor aortic patch was connected to recipient aorta in an end-to-side manner. Urinary reconstruction was performed by ureter-to-bladder anastomosis. The total surgical ischemia time was restricted to <45 min.

Animal models were as follows: Lewis–Lewis isografts short-term: the contralateral kidney was removed immediately after surgery. Grafts were harvested 24 h after transplantation, at which the delayed graft function (DGF) occurs.

Lewis–Lewis isografts and Fischer–Lewis allografts long-term survival: The contralateral kidney was removed 4 days after surgery to enable the animal to survive through the acute DGF period. Fischer-Lewis allografts received once daily intramuscular doses of cyclosporine A in 5 mg/kg for 10 days. All animals were monitored on a daily basis with a scoring assay based on body weight, activity, general appearance and behavior 14. Any animal that scored over 7 was killed.

### Gas exposure *in vivo*

Rats were exposed to either 70% xenon or 70% nitrogen balanced with 30% O_2_ for 2 h via an anesthetic chamber. Gas was given either to donor 24 h prior to organ retrieval (donor pretreatment), or immediately after grafting (recipient posttreatment). Gas concentrations of xenon and oxygen were constantly monitored by xenon monitor (Air Products Model No. 439 Xe, Bradford, UK) and Datex monitor (Datex-Ohmeda, Bradford, UK).

### Hydrodynamic tail vein injection

HIF-1α siRNA or scrambled siRNAs (negative control) (Qiagen) were dissolved in siRNA suspension buffer and further diluted in RNase-free PBS before use. siRNA targeting rat HIF-1α (Sense strand; 5′-GGAAACGAGUGAAAGGAUATT-3′, Antisense strand: 5′-UAUCCUUUCACUCGUUUCCAA-3′) was administered through Hydrodynamic tail vein injection. HIF-1α or scrambled siRNA (200 µg in 10 ml of PBS) was rapidly injected (within 30 s) via a tail vein under anesthesia and allow to recover for 24 h before xenon treatment.

### Hematoxylin and eosin staining

The kidney graft was fixed in 4% paraformaldehyde and embedded with paraffin. Sections of 5-µm thickness were taken and hematoxylin & eosin (H&E) staining was carried out.

### Immunohistochemistry

Kidney sections were incubated with goat anti-TLR-4 (1:200, Santa Cruz Biotechnology, Dallas, TX), rabbit anti-cleaved caspase-3 (1:200, Cell Signalling, Danvers, MA), rabbit anti-NF-κB p65 (1:200, Abcam), rabbit anti-cleaved caspase-3 (1:400, Cell Signalling, Danvers, MA), HK-2 cells were incubated with rabbit anti-HIF-1α (1:200, Novus, Littleton, CO), mouse anti-VEGF (1:200, Abcam), rabbit anti-Bcl-2 (1:200, Abcam), rabbit anti-HMGB-1 (1:200, Abcam), mouse anti- α-tubulin (1:200, Sigma–Aldrich, UK), followed by fluorescently-conjugated secondary antibodies. Slides were counterstained with DAPI. For DAB staining, kidney paraffin sections were incubated with rabbit anti-rat CD3 (1:200, Abcam) and then with biotin-conjugated secondary antibody. The slides were visualized with 3,3-diaminobenzidine tetrahydrochloride (DAB) followed by counterstaining with hematoxylin. Immunofluorescence was quantified using Image J (National Institutes of Health, Bethesda, MD). Ten high-power fields at 20× magnification were photographed. Fluorescent intensity was calculated as percentages of the mean value for naïve controls.

### Western blotting

The tissue lysates from kidney samples or cultured cells were centrifuged and the protein extracts (40 µg/sample) underwent electrophoresis and then transferred to a PVDF membrane. The membrane was treated with blocking milk solution and probed with rabbit anti-PI-3K (1:1000, Cell Signalling), rabbit anti-phospho-Akt (1:1000, Cell Signalling), rabbit anti-phospho-mTOR (1:1000, Cell Signaling), rabbit anti-HIF-1α (1:1000, Novus), rabbit anti-Caspase-3 (Cleaved, 1:1000, Cell Signalling), rabbit anti-p65 NF-κB (1:1000, Abcam), goat-anti-TLR-4 (1:1000, Santa Cruz Biotechnology), mouse anti-VEGF (1:1000, Abcam) and rabbit anti-Bcl-2 (1:1000 Abcam), followed by HRP-conjugated secondary antibody. The loading control was α-tubulin (1:10 000, Sigma–Aldrich). The blots were visualized with enhanced chemiluminescence (ECL) system (Santa Cruz Biotechnology) and analyzed with GeneSnap (Syngene, Cambridge, UK). Protein band intensity was nomalized with α-tubulin and expressed as ratio of control.

### Enzyme-linked immunosorbent assay (ELISA)

Rat TNF-α, IL-1β, IL-6, and HMGB-1 were measured by ELISA (Rat TNF-α, IL-1β, IL-6 ELISA kit, Invitrogen, rat HMGB-1 ELISA kit, BL International, Hamburg, Germany).

### Renal function

Blood samples were collected when animals were killed. After centrifugation, serum urea and creatinine concentrations were measured using an Olympus AU2700 analyzer (Diamond Diagnostics, Watford, UK).

### Statistical analysis

All numerical data were expressed as mean ± standard deviation (SD). Comparison between treatment groups was analyzed by one-way ANOVA followed by *post hoc* Student Newman–Keuls test (GraphPad Prism 5.0 Software). Animal survival analysis was performed using Kaplan–Meier survival estimates, and statistical significance was analyzed by the log-rank test (GraphPad Prism 5.0; GraphPad Software). A p value <0.05 was considered to be a statistical significance.

## Results

### Xenon treatment enhanced the expression of HIF-1α, VEGF and Bcl-2 in HK-2 cells

**Figure 1:**
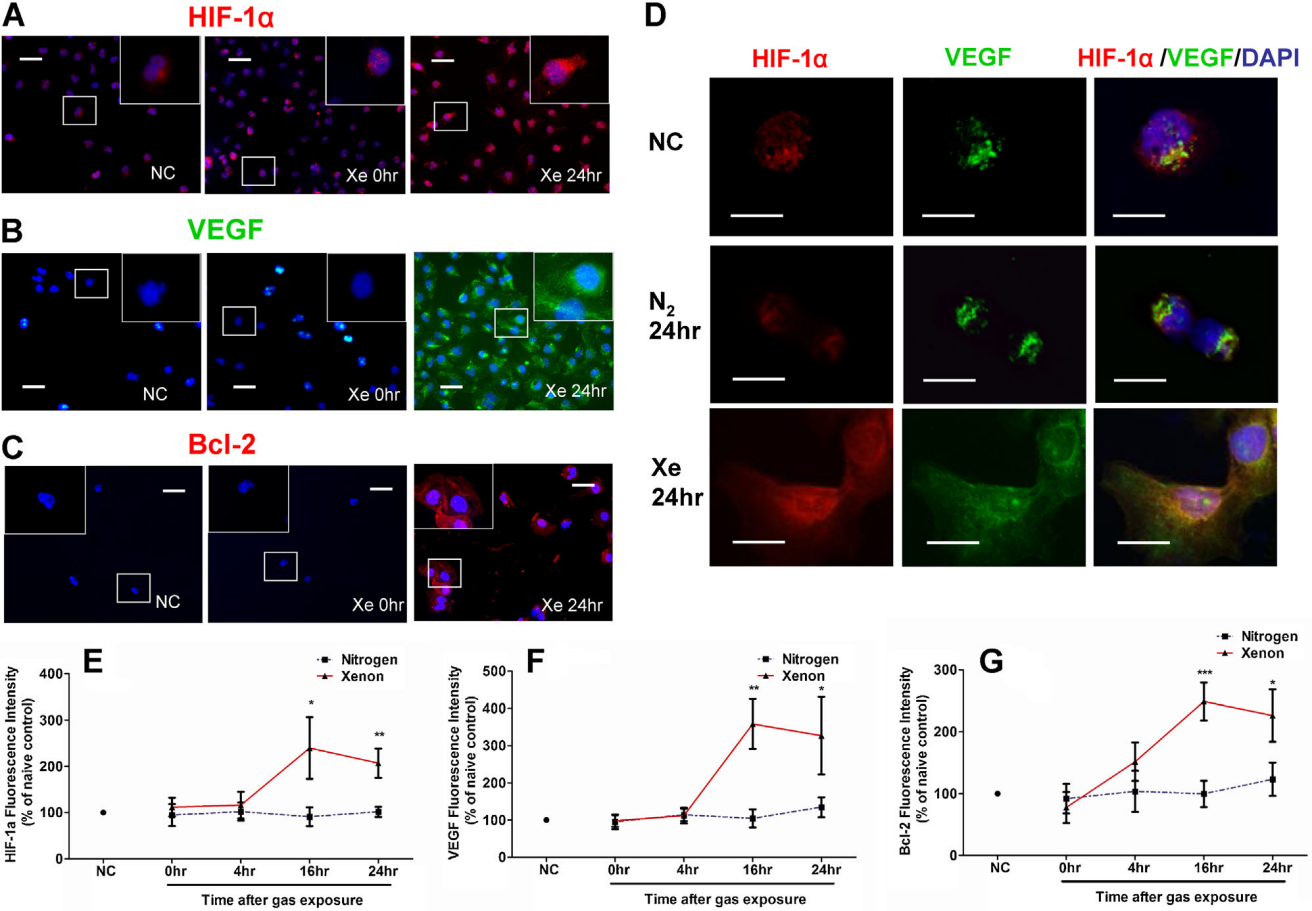
HIF-1α, VEGF and Bcl-2 expression in human kidney proximal tubular cells (HK-2) induced by xenon (Xe) exposure. HK-2 cells were treated with 70% Xe and 5% CO_2_ balanced with O_2_ for 2 h and then recovered in the normal cell incubator for 24 h. HIF-1α (Red; A), VEGF (Green; B), and Bcl-2 immunoreactivity (Red; C) are shown in naïve control and 0–24 h after gas exposure. (D) Immunofluoresent dual-labeling of HIF-1α and VEGF on HK-2 cells treated with xenon. (Top panel) Naïve control (NC), normal HK-2 cells exposed to normal air. Middle panel: nitrogen exposure, HK-2 cells were treated with nitrogen gas (70% N_2_ and 5% CO_2_ balanced with O_2_) for 2 h and then recovered in the normal cell incubator for 24 h. Bottom panel: HK-2 cells were treated with xenon gas (70% Xe and 5% CO_2_ balanced with O_2_) for 2 h and then recovered in the normal cell incubator for 24 h, HIF-1α (Red) and VEGF (Green) co-expressed on xenon-treated cells 24 h after gas exposure. Scale bar: 50 μm. Mean fluorescence intensity (% of naïve control) of HIF-1α (E), VEGF (F), and Bcl-2 (G) after gas exposure shows the expression levels of these proteins were significantly increased 16 h after Xe exposure when compared with N_2_ control. NC, naïve control. Bars represent means ± SD (n = 4). *p < 0.05, **p < 0.01 and ***p < 0.001 Xe versus N_2_ treatment. Scale bar: 50 μm.

Hypoxia inducible factor-1α (HIF-1α), vascular endothelial growth factor (VEGF) and Bcl-2 in human proximal tubular cell line (HK-2) were measured via immunofluorescent staining after exposure to 70% Xe and 5% CO_2_ balanced with O_2_ for 2 h (Figure 1). HIF-1α expression was increased 16 h after gas exposure (Figure 1A and E). Similarly, its downstream effector VEGF was enhanced (Figure 1B and F). A significant increase in Bcl-2 expression was observed 16 h after gas exposure (Figure 1C and G). Co-localization of HIF-1α and VEGF was evident in HK-2 cells 24 h after exposure to xenon gas (Figure 1D). These data indicated that xenon exposure was associated with the downstream activation of pro-survival proteins which enhance the cell survival.

### Xenon treatment preserved cell structural integrity and reduced the cellular inflammation after hypothermia–hypoxia challenge

**Figure 2:**
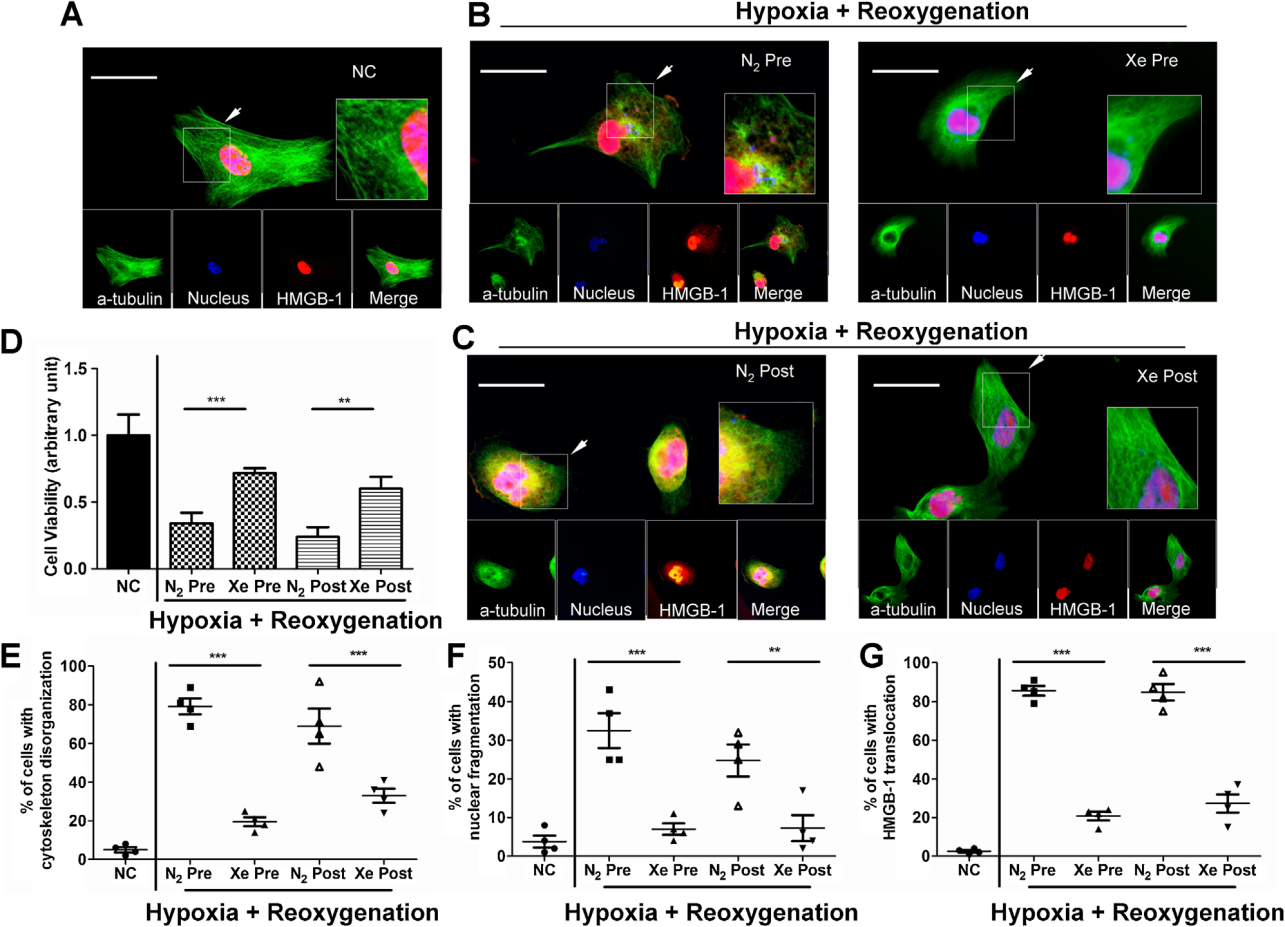
Xenon attenuated nuclear fragmentation, cytoskeletal disorganization and HMGB-1 translocation of HK-2 cells after hypothermia-hypoxia challenge. HK-2 cells were given 24 h hypothermia-hypoxia challenge (4°C soltran preserving solution under 8% O_2_) and then followed by 24 h reoxygenation (37°C culture medium in normal cell incubator). Two hours gas exposure (70% Xe or N_2_, and 5% CO_2_ balanced with O_2_) was given either 24 h prior to hypothermia–hypoxia challenge (pretreatment) or immediately after hypothermia–hypoxia challenge (posttreatment). (A–C)The cytoplasm and nucleus were immunolabeled with anti α-tubulin (Green) and DAPI nuclear stain (Blue). HMGB-1 (Red) translocated from nucleus to cytoplasm in nitrogen-treated groups, while xenon treatment restricted the translocation. Scale bar: 50 μm. (D) Cell viability after hypoxia-reoxygenation was assessed by MTT and the ratio against the NC was used as the arbitrary unit. Percent of HK-2 cells with cytoskeleton disorganization (E), nuclear fragmentation (F), HMGB-1 cytoplasmic translocation (G). Data are means ± SD (n = 4), significance level is shown as **p < 0.01 and ***p < 0.001. NC, naïve control.

Extracellular high-mobility group box-1 (HMGB1) is a potent innate “danger signal” for the initiation of inflammatory response. Cellular stress and injury during ischemia-reperfusion normally causes necrotic nuclear disintegration, translocation of HMGB-1 into the cytoplasm and its subsequent release into extracellular space. Rapid repair and replication promoted by persistent HIF-1α up-regulation could restore the integrity of cellular structures and contain HMGB-1 within the nucleus, thus preventing the activation of innate immunity in the extracellular milieu. To determine if xenon could prevent release of HMGB-1, we performed an immunofluorescence analysis of HMGB-1 and cellular structural changes. HMGB-1 in naïve HK-2 cells is universally distributed inside the nucleus (Figure 2A). Exposure to nitrogen gas (70% N_2_, 5% CO_2_ balanced with O_2_) did not alter the cytoplasmic translocation of HMGB-1 after hypothermia-hypoxia challenge but surprisingly, with xenon (70% Xe, 5% CO_2_ balanced with O_2_) pretreatment, HMGB-1 remained to a large extent within the nuclei (Figure 2B) after hypothermia-hypoxia challenge. In addition, a similar effect with xenon posttreatment to HK-2 cells was found with HMGB-1 restricted to the nucleus in the majority of the cells (Figure 2C). Notably, in contrast to naïve controls, the nitrogen treated cells given a hypothermia-hypoxia challenge showed more chaotic distribution of cytoskeleton network structure; while HK-2 cells treated with xenon before or after hypothermia-hypoxia challenge tended to have a more organized cytoskeletal network and better integrity of the nuclei, which correlates well with nuclear HMGB-1 localization (Figure 2E–G). The HK-2 cell viability after insults was enhanced after xenon exposure (Figure 2D).

### Xenon induced renoprotective protein expression in renal cortex

**Figure 3:**
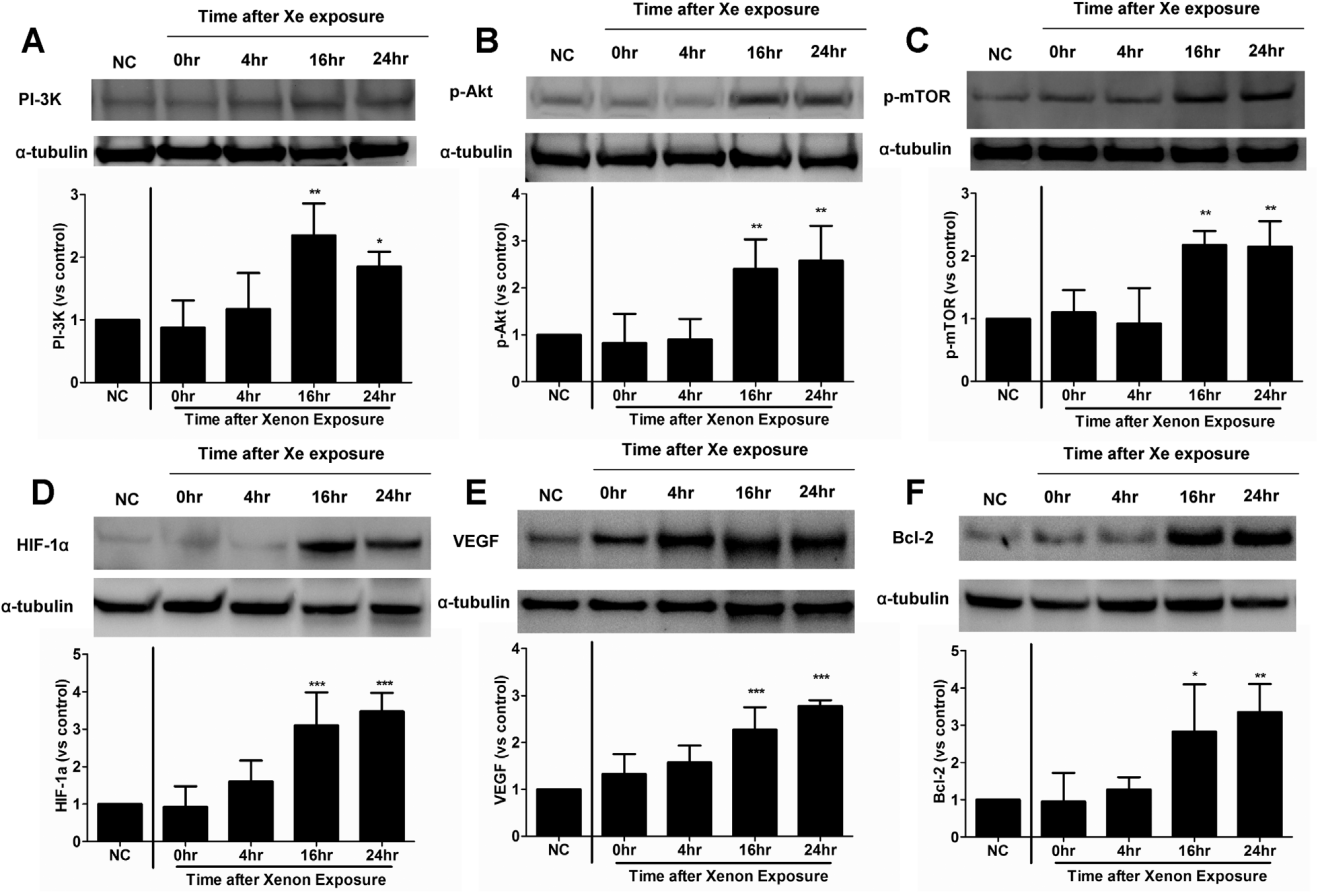
Activation of PI3K-Akt-mTOR pathway, and enhanced expression of HIF-1α, VEGF and Bcl-2 expression on renal cortex by xenon gas. Lewis rats were exposed to xenon gas (70% Xe balanced with 30% O_2_) for 2 h and then room air for 24 h. Expressions of PI-3K (A), p-Akt (B), p-mTOR (C), HIF-1α (D), VEGF (E), and Bcl-2 (F) were assessed by Western blot at 0–24 h after gas exposure. Normal kidney graft serves as naïve control (NC). PI3K-AKT-mTOR pathways were activated 16 h after gas exposure compared with normal control and significant up-regulation of HIF-1α, VEGF and Bcl-2 was found 16 h after gas exposure compared naive control. Data are means ± SD (n = 4). *p < 0.05, **p < 0.01 and ***p < 0.001. Xe exposure versus naïve control.

To answer the question of whether the PI3K-Akt-mTOR pathway is transmitting cell survival and proliferation signaling in renal tubular cells in vivo, the expression levels of PI-3K, phospho-Akt and phospho-mTOR in the kidney were examined (Figure 3A–C). Animals were exposed to 70% Xe balanced with 30% O_2_. Western blot analysis revealed a significant increase in this pathway in the renal cortex compared with naive control 16 h after gas exposure. PI-3K was increased up to twofold relative to the naïve control (2.35 ± 0.5 vs. 1.00 ± 0.01 of control, p < 0.01) and both p-Akt and p-mTOR were increased nearly three times (p-Akt 2.4 ± 0.63 vs. 1.00 ± 0.02 of control, p < 0.01, and p-mTOR 2.18 ± 0.22 vs. 1.00 ± 0.02 of control, p < 0.01). The up-regulation of these proteins was maintained up to 24 h post-exposure (p < 0.05 for PI-3k, p < 0.01 for p-Akt and p-mTOR respectively, at 24 h vs. control). To confirm whether the expression of Akt-mTOR pathway leads to productions of HIF-1α and Bcl-2, the expression patterns of HIF-1α, VEGF and Bcl-2 were evaluated (Figure 3D–F). Only basal levels of HIF-1α and Bcl-2 expression were detected in naive kidneys. The expression of both HIF-1α and Bcl-2 surged to high level in the renal cortex 16 h after gas exposure, compared with the naive control (HIF-1α 3.1 ± 0.88 vs. 1.00 ± 0.02 of control, p < 0.001 and Bcl-2 2.8 ± 1.26 vs. 1.00 ± 0.02 of control, p < 0.05). The expression of VEGF increased gradually after the gas treatment, peaking at 16 h post-exposure (2.27 ± 0.48 vs. 1.00 ± 0.02 of control, p < 0.001). Expression of all these proteins remained consistently enhanced at 24 h postexposure (p < 0.01 vs. control).

### Xenon treatment prevented translocation of HMGB-1 and activation of TLR-4 in kidney graft after transplantation

**Figure 4:**
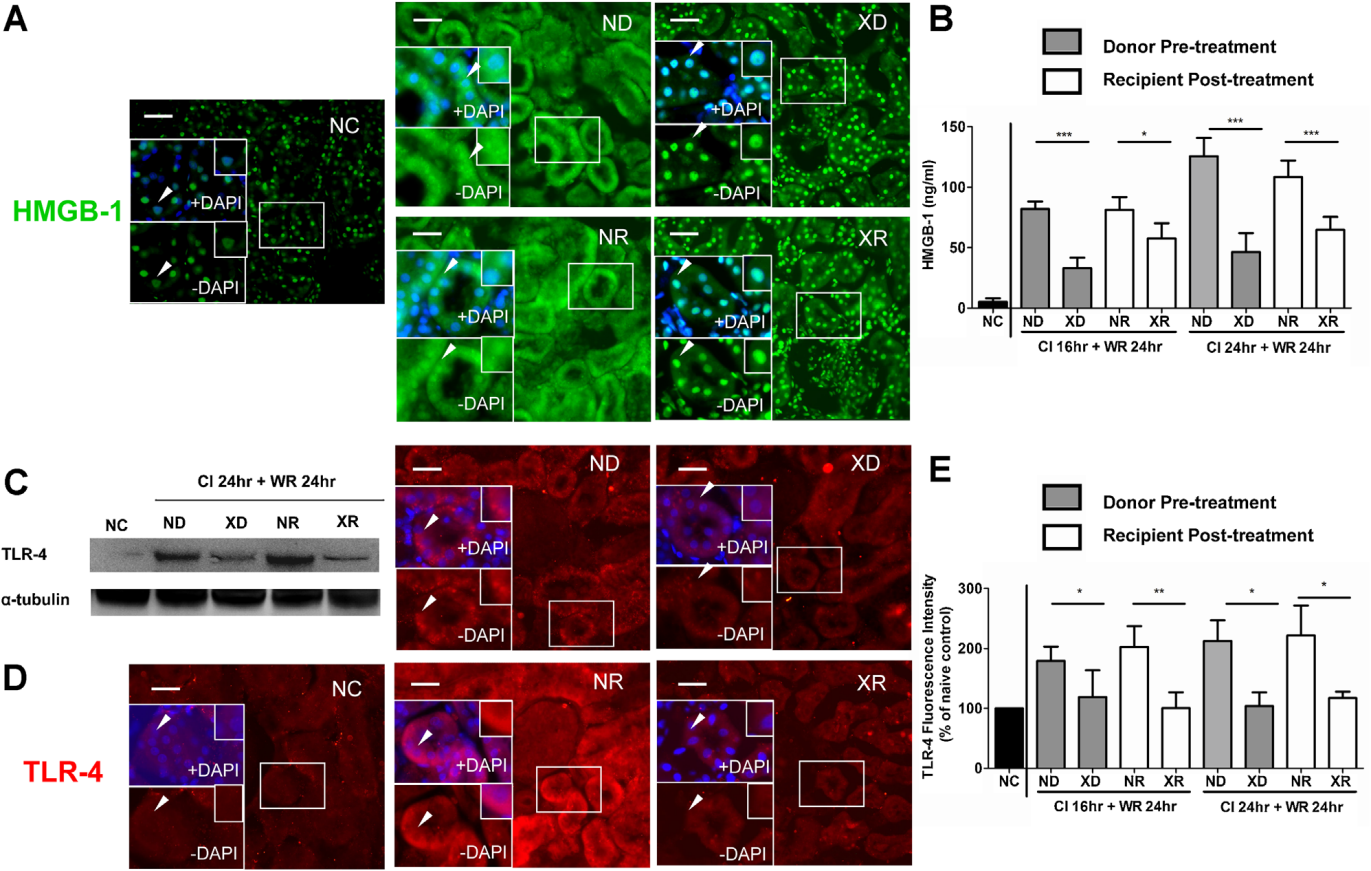
HMGB-1 and TLR-4 expression in cortical tubular cells after engraftment. The Lewis renal graft was stored in 4°C Soltran preserving solution for 16 h (Cold Ischemia CI 16 h) or for 24 h (Cold Ischemia CI 24 h) and then transplanted into Lewis recipient, the graft was harvested 24 h after transplantation (Warm Reperfusion, WR 24 h). The xenon gas (70% Xe balanced with 30% O_2_) was given either to donor (XD, Xenon-Donor treatment) 24 h before donor organ retrieval or given to recipient (XR, Xenon-Recipient treatment) immediately after organ engraftment. Animals receiving nitrogen gas (70% N_2_ balanced with 30% O_2_) in donor stage (ND, Nitrogen-Donor treatment) or recipient (NR, Nitrogen-Recipient treatment) served as treatment control. (A) Both xenon donor and xenon recipient treatment prevented the cytoplasmic release of HMGB-1 (green fluorescence) on cortical tubules after 24 h CI and 24 h WR compared with control grafts. Scale bar: 50 μm. (B) Serum HMGB-1 concentration of the recipient assessed by ELISA. Data is expressed as mean ± SD (n = 4). (C and D) Both xenon donor and xenon recipient treatment reduced the expression of TLR-4, compared with control grafts, demonstrated by Western blotting and immunofluorescence (red) on cortical tubules. Scale bar: 50 μm. (E) Mean fluorescence intensity of TLR-4 in renal cortical tubules (% of naïve control). NC, naïve control. Both xenon donor and xenon recipient treatment reduced the concentration of pro-inflammatory cytokines compared with nitrogen control grafts (*p < 0.05, **p < 0.01 and ***p < 0.001).

To further confirm the *in vitro* finding shown (Figure 2), the effects of xenon on the release of HMGB-1 were also examined *in vivo*. Twenty-four hours after renal transplantation, HMGB-1 was translocated from the nucleus into the cytosol, and then secreted into the extracellular space making cellular structure become undistinguishable after up to 24 h cold ischemia. Both donor and recipient xenon treatments stabilized and preserved the integrity of tubular cell nuclei and decreased the translocation of HMGB-1 into the cytoplasm (Figure 4A). Consistent with these findings, serum HMGB-1 was also decreased with xenon donor (N_2_ vs. Xe in CI 24 h: 125.5 ± 15.1 ng/ml vs. 46.4 ± 15.5 ng/ml, p < 0.01) and with xenon recipient (N_2_ vs. Xe in CI 24 h: 108.3 ± 13.6 ng/ml vs. 64.7 ± 10.6 ng/ml, p < 0.01) treatment (Figure 4B). Release of DAMP such as HMGB-1 is associated with inflammatory response, this response is mediated via a signaling cascade which is TLR-4 dependent. Immunofluorescent studies indicated that naive kidney tissue expressed TLR-4 at a basal level. Twenty-four hours after grafting, kidney grafts subjected to 16 or 24 h ischemia and receiving nitrogen gas displayed high immunoreactivity for TLR-4, notably on the surface of renal tubules and infiltrating leukocytes. Both xenon donor and recipient treatment decreased TLR-4 expression (Figure 4D and E), and this result was verified through western blotting of renal graft subjected to 24 h cold ischemia followed by 24 h reperfusion (Figure 4C).

### Xenon treatment decreased NF-κB and caspase-3 activation in kidney graft after transplantation

**Figure 5:**
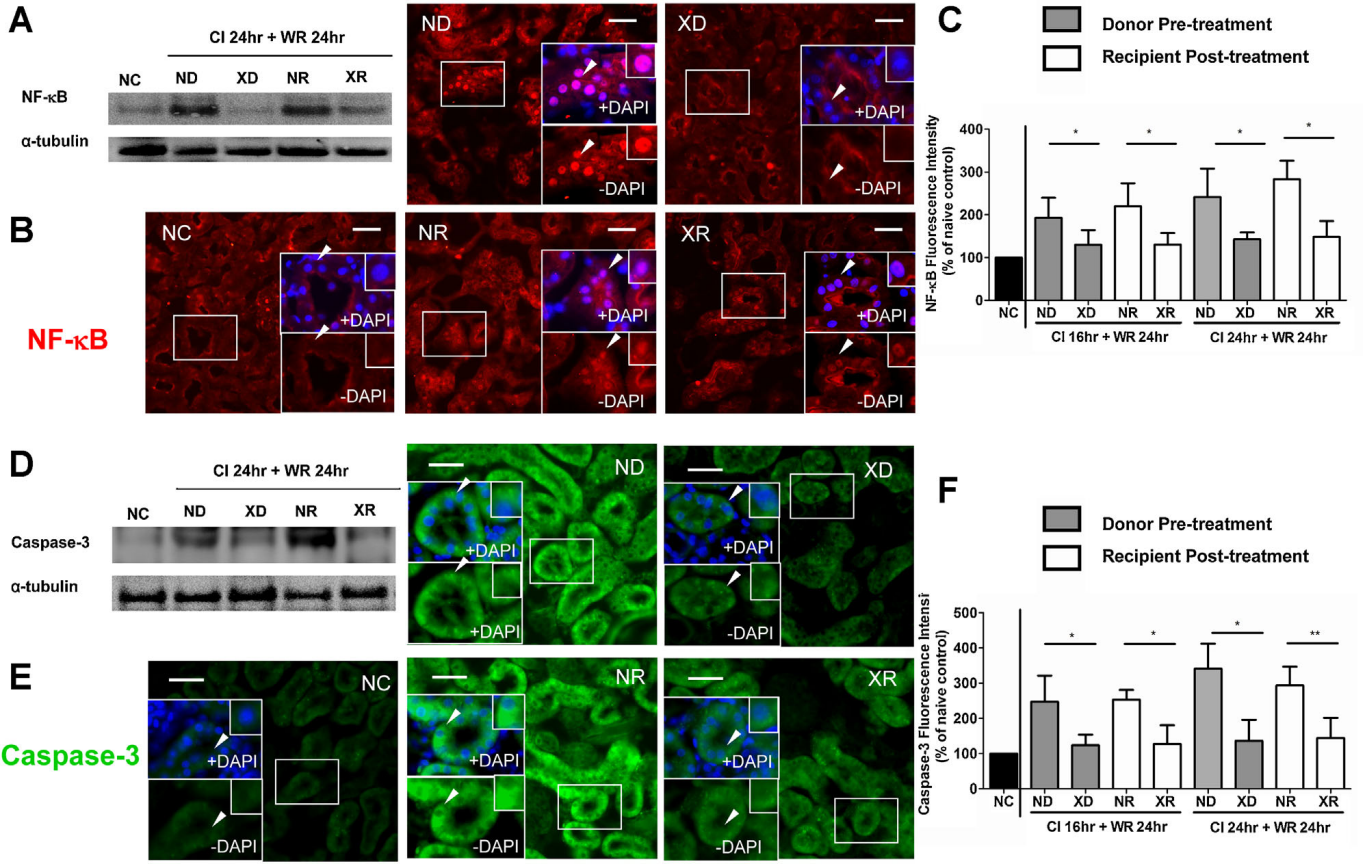
NF-κB and caspase-3 expression in cortical tubular cells after engraftment. The Lewis renal graft was stored in 4°C Soltran preserving solution for 16 h (Cold ischemia CI 16 h) or for 24 h (cold ischemia CI 24 h) and then transplanted into Lewis recipient, the graft was finally harvested 24 h after transplantation (Warm Reperfusion, WR 24 h). The xenon gas (70% Xe and 30% O_2_) was given either to donor (XD, Xenon-Donor treatment) 24 h before donor organ retrieval or given to recipient (XR, Xenon-Recipient treatment) immediately after organ engraftment. Animals receiving nitrogen gas (70% N_2_ balanced with 30% O_2_) in donor stage (ND, Nitrogen-Donor treatment) or recipient (NR, Nitrogen-Recipient treatment) served as treatment control. (A and B) Both xenon donor and xenon recipient treatment reduced the expression of NF-κB, compared with control grafts, demonstrated by western blotting and immunofluorescence (red) on cortical tubules. Scale bar: 50 μm. (C) Mean fluorescence intensity of NF-κB in renal cortical tubules (% of naïve control). (D and E) Both xenon donor and xenon recipient treatment reduced the expression of caspase-3, compared with control grafts, demonstrated by Western blot and immunefluorescence (green) on cortical tubules. Scale bar: 50 μm. (F) Mean Fluorescence intensity of caspase-3 in renal cortical tubules (% of naïve control). NC, naive control. Data is expressed as mean ± SD (n = 4, *p < 0.05 and **p < 0.01).

We next examined the level of tubular apoptosis and inflammation after ischemia-reperfusion in the Lewis-to-Lewis renal transplantation model. The grafts underwent 16 or 24 h of cold ischemia and were harvested at Day 1 after transplantation. Signaling through TLR-4 increases NF-κB activity which is essential for induction of pro-inflammatory cytokines genes 15. As shown in Figure 5B and C, in the naïve kidney, cytoplasmic expression of NF-κB was maintained at a minimal basal level. In nitrogen treated animals, activated NF-κB was detected mainly in cortical tubular epithelial cells and sparsely in some glomerular cells. Both xenon donor or xenon recipient treatment decreased the NF-κB signaling intensity, in contrast, with nitrogen treatment, NF-κB expression was enhanced and translocation into nuclei was intensified. The xenon-induced decrease of NF-κB expression was confirmed by Western blot study on grafts with 24 h cold ischemia followed by 24 h warm reperfusion (Figure 5A). Caspase-3 immunoreactivity was measured in the renal cortex, which is involved in execution of apoptosis. Xenon treatment of donor or recipient significantly decreased the intensity of cytoplasmic staining compared with nitrogen treatment (Figure 5E and F). Reduction of caspase-3 and expression was confirmed by Western blot analysis of grafts with 24 h cold ischemia followed by 24 h warm reperfusion (Figure 5D).

### Xenon treatment decreased proinflammatory cytokine release and prevented delayed graft function (DGF) after kidney engraftment

**Figure 6:**
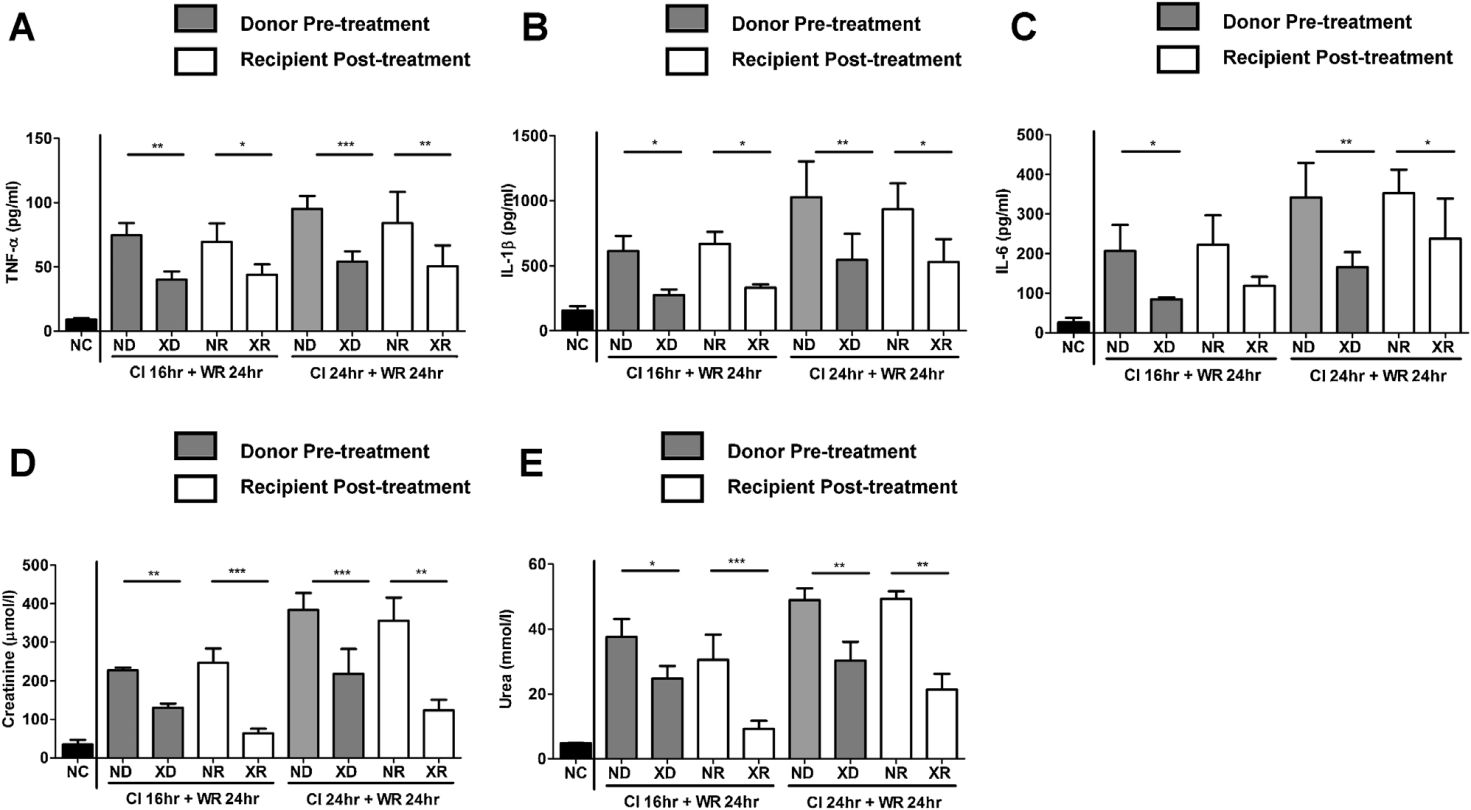
Serum TNF-α, IL-1β and IL-6, serum creatinine and urea concentration after engraftment. The Lewis renal graft was stored in 4°C Soltran preserving solution for 16 h (Cold Ischemia CI 16 h) or for 24 h (Cold Ischemia CI 24 h) and then transplanted into Lewis recipient, the graft was finally harvested 24 h after transplantation (Warm Reperfusion, WR 24 h). The xenon gas (70% Xe and 30% O_2_) was given either to donor (XD, Xenon-Donor treatment) 24 h before donor organ retrieval or given to recipient (XR, Xenon-Recipient treatment) immediately after organ engraftment. Animals receiving nitrogen gas (70% N_2_ balanced with 30% O_2_) in donor stage (ND, Nitrogen-Donor treatment) or recipient (NR, Nitrogen-Recipient treatment) served as treatment control, normal kidney serves as naïve control (NC; A-C) Serum cytokine TNF-α, IL-1β and IL-6 concentration was assessed by ELISA. Data is expressed as mean ± SD (n = 4). (D and E) Serum creatinine and urea concentration were analyzed and expressed as Mean ± SD (n = 4). Both xenon donor and xenon recipient treatment reduced the concentration of pro-inflammatory cytokines and restore renal function compared with nitrogen control grafts (*p < 0.05, **p < 0.01 and ***p < 0.001).

Serum levels of proinflammatory cytokines, including IL-1β, TNF-α and IL-6, were reduced by xenon treatment to donor or to recipient but not affected by nitrogen treatment (Figure 6A–C). We then evaluated the effect of xenon treatment on graft function after severe ischemia-reperfusion. The function of kidney grafts receiving nitrogen gas before or after ischemia deteriorated rapidly, treatment with xenon preserved renal function after grafting (Figure 6D and E).

### HIF-1α siRNA attenuated xenon mediated protection

**Figure 7:**
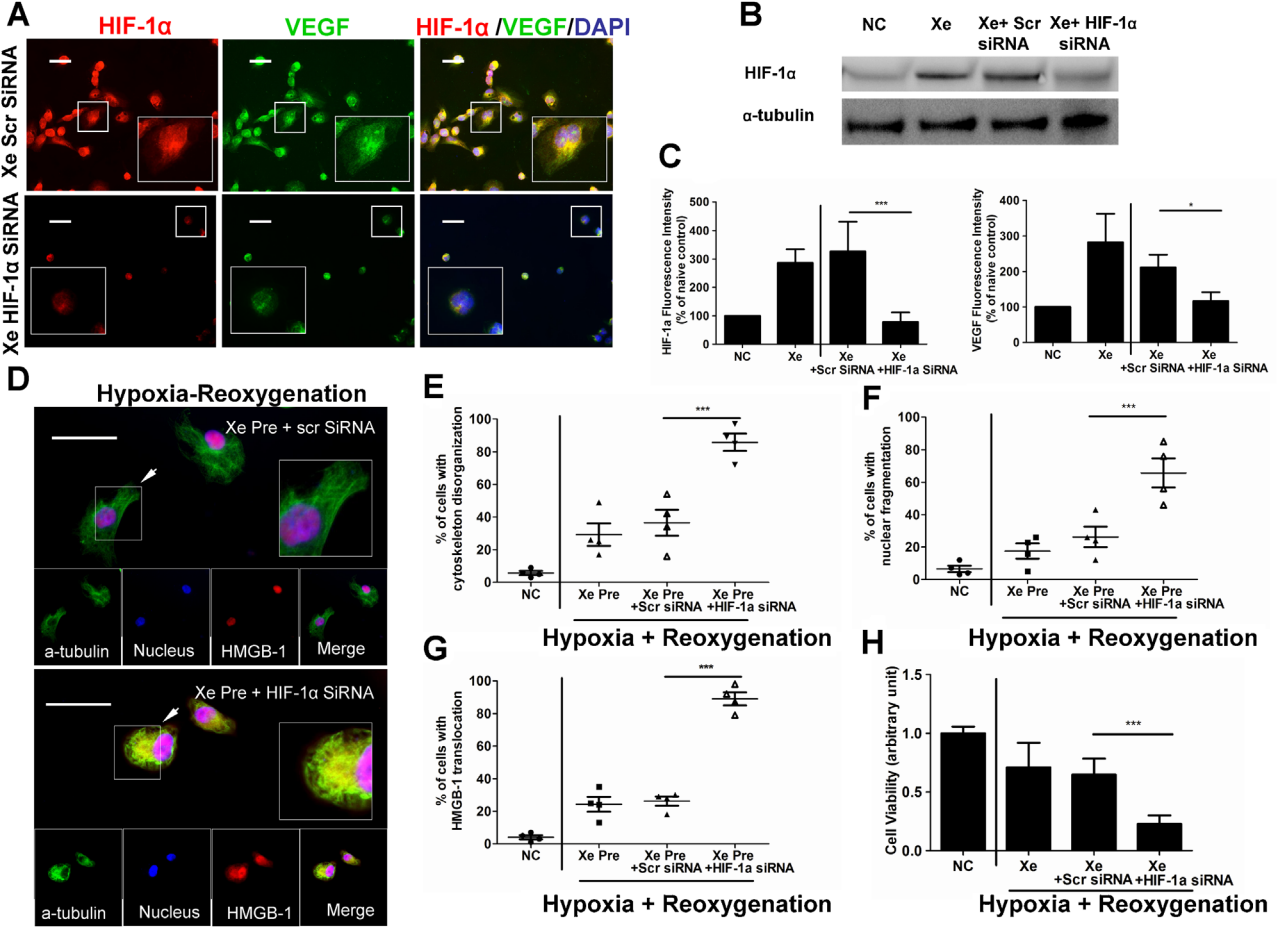
Effect of HIF-1α siRNA on the xenon mediated cytoprotection. HK-2 cells were treated with either scrambled siRNA or HIF-1α siRNA (20 nM) for 6 h, followed by xenon gas (70% Xe and 5% CO_2_ balanced with O_2_) for 2 h and then recovered in normal cell incubator for 24 h. (A) Immunoluorescent dual-labeling of HIF-1α (Red) and VEGF(Green) in HK-2 cells. Scale bar: 50 μm. (B) Knock-down of HIF-1α was confirmed by Western blot. (C) Mean fluorescence intensity of HIF-1α and VEGF after HIF-1α siRNA treatment. The cells underwent 24 h hypothermia-hypoxia challenge and recovered (reoxygenation) for 24 h. (D) Effect of xenon on HMGB-1 (Red) cytoplasmic translocation, was abolished by HIF-1α siRNA treatment. Scale bar: 50 μm. Percent of HK-2 cells with cytoskeleton disorganization (E), nuclear fragmentation (F) and HMGB-1 cytoplasmic translocation (G) per 20× images. (H) Cell viability was assessed by an MTT assay. Data is expressed as mean ± SD (n = 4); *p < 0.05 and ***p < 0.001.

**Figure 8:**
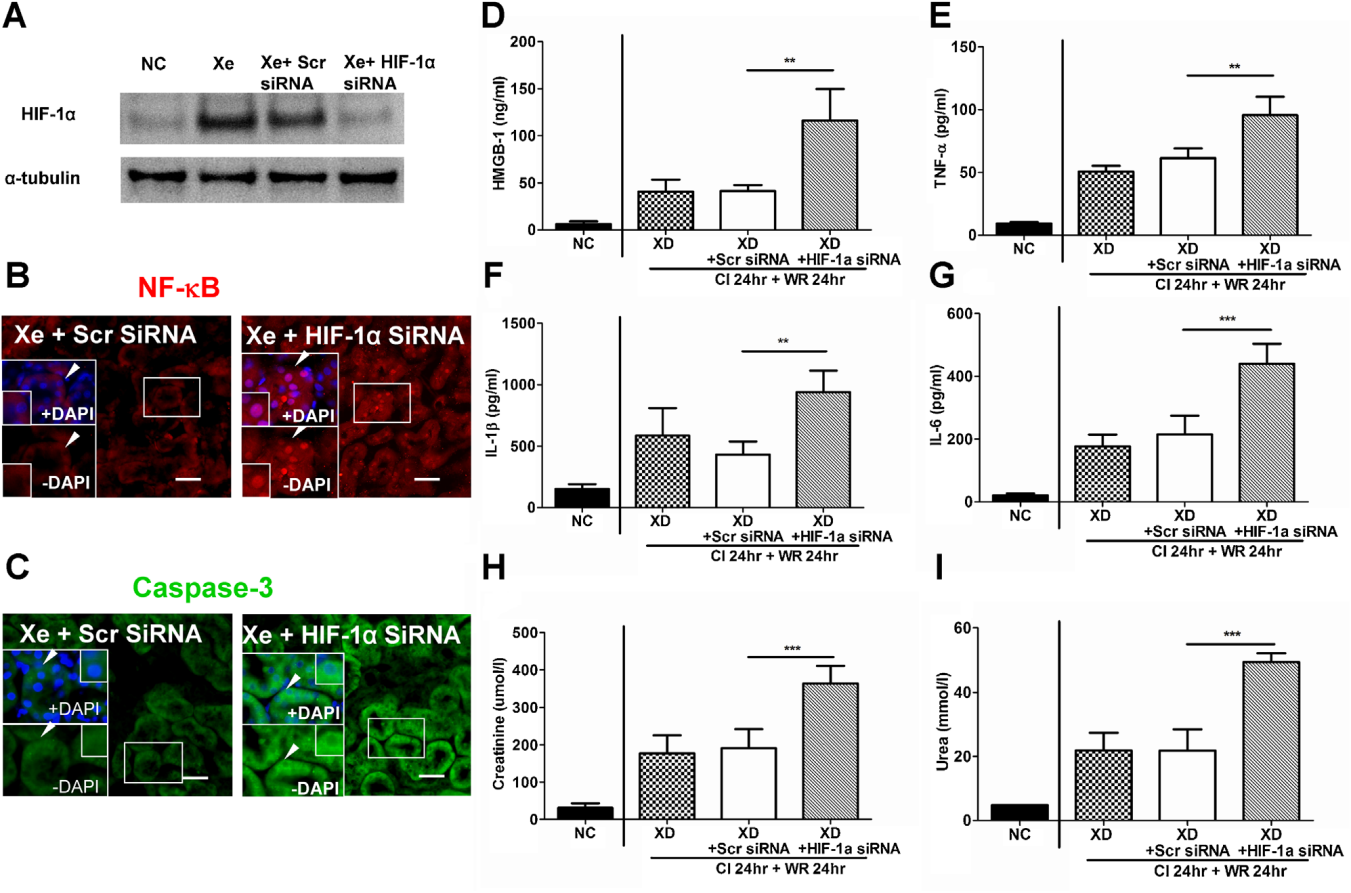
HIF-1α siRNA abolished renoprotection mediated by xenon in renal grafts. Donor Lewis rats were treated with scrambled siRNA or HIF-1α siRNAs, 24 h after injection, then followed by xenon pretreatment. The grafts were stored in cold preserving solution for 24 h and transplanted into Lewis recipient (cold ischemia CI 24 h), 24 h after transplantation, the blood sample of the recipient was analyzed (Warm reperfusion WR, 24 h). (A) HIF-1α siRNA abolished HIF-1α up-regulation induced by xenon. At 24 h after grafting, expression of NF-κB (B) and caspase-3 (C) were markedly enhanced in Xe- HIF-1α siRNA treated graft. (D–G) Serum HMGB-1, TNF-α, IL-1β and IL-6 levels in Xe HIF-1α siRNA-treated rats were significantly higher than in Xe scrambled siRNA-treated rats (n = 3). (H and I) Serum creatinine and urea levels in Xe HIF-1α siRNA-treated rats were significant higher than in Xe scrambled siRNA-treated rats (n = 3). Data is expressed as mean ± SD, **p < 0.01 and ***p < 0.001.

To further confirm the role of HIF-1α on xenon mediated protection, 20 nM of HIF-1α siRNA was administered to HK-2 cells followed by xenon pretreatment (Figure 7). Compared with the scramble siRNA, HIF-1α siRNA administration attenuated the up-regulation of HIF-1α and VEGF induced by xenon treatment (Figure 7A–C). After hypothermia-hypoxia challenge, considerably high level of cytoplasmic staining of HMGB-1 was detected with HIF-1α siRNA treatment compared with that with scramble siRNA, together with increased nuclear and cytoskeleton damages (Figure 7D–G). Enhanced cell viability by xenon treatment was abolished by HIF-1α siRNA treatment after hypothermia–hypoxia challenge (Figure 7H). These observations were also supported by *In vivo* data obtained by using the hydrodynamic injection of HIF-1α techniques (Figure 8A). Enhanced NF-κB expression (Figure 8B) and Caspase-3 (Figure 8C) expression were found in HIF-1α siRNA treated grafts. The anti-inflammatory effect of xenon was abolished with HIF-1α siRNA injection (Figure 8D–G). Grafts received HIF-1α siRNA developed delayed graft function, with high level of creatinine and urea (Figure 8H and I).

### Donor xenon pretreatment and recipient posttreatment prolonged the survival of isografts and allografts

**Figure 9:**
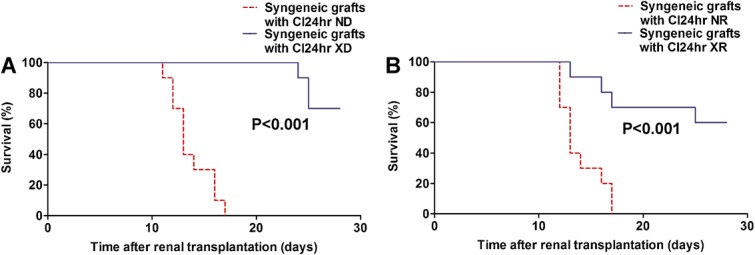
Xenon treatment prolonged the syngeneic grafts survival. Xenon treatment (70% xenon balanced with 30% oxygen) was given either to Lewis donor animals before organ retrieval (XD) or Lewis recipient immediately after engraftment (XR). Grafts were kept in 4°C Soltran preserving solution (cold ischemia; CI) for 24 h. Donor or recipient exposed to nitrogen (70% N_2_ balanced with 30% O_2_) served as treatment control. Contralateral kidney was removed 4 days after transplant surgery. Kaplan–Meier survival analysis of recipient animals in Lewis-to-Lewis isografts. Xenon gas was given to donor Lewis animals (A) before organ retrieval or to recipient immediately (B) after engraftment (n = 10).

The survival of animals receiving isografts that were stored at 4°C in Soltran for 24 h was significantly prolonged by pretreatment with xenon, as compared with nitrogen treated groups (Figure 9). All nitrogen treated groups (whether to donor or to recipient) developed signs of acute renal failure within 3 weeks posttransplant. The survival for xenon donor group was significantly greater than that for the N_2_ donor group (p < 0.001; Figure 9A) A similar pattern of survival was found in the posttreatment to recipients (Figure 9B; p < 0.001, Xe vs. N_2_).

In the Fischer to Lewis allogeneic transplantation, Fisher kidney grafts were stored in 4°C Soltran solution for 16 h. Xenon treatment preserved renal morphology on Day 20 after transplantation (Figure 10A). CD3^+^ T cell infiltration was significantly reduced by xenon treatment on Day 20 after surgery (Figure 10B). The allograft survival was significantly prolonged by xenon donor or recipient treatment (Figure 10C and D).

**Figure:**
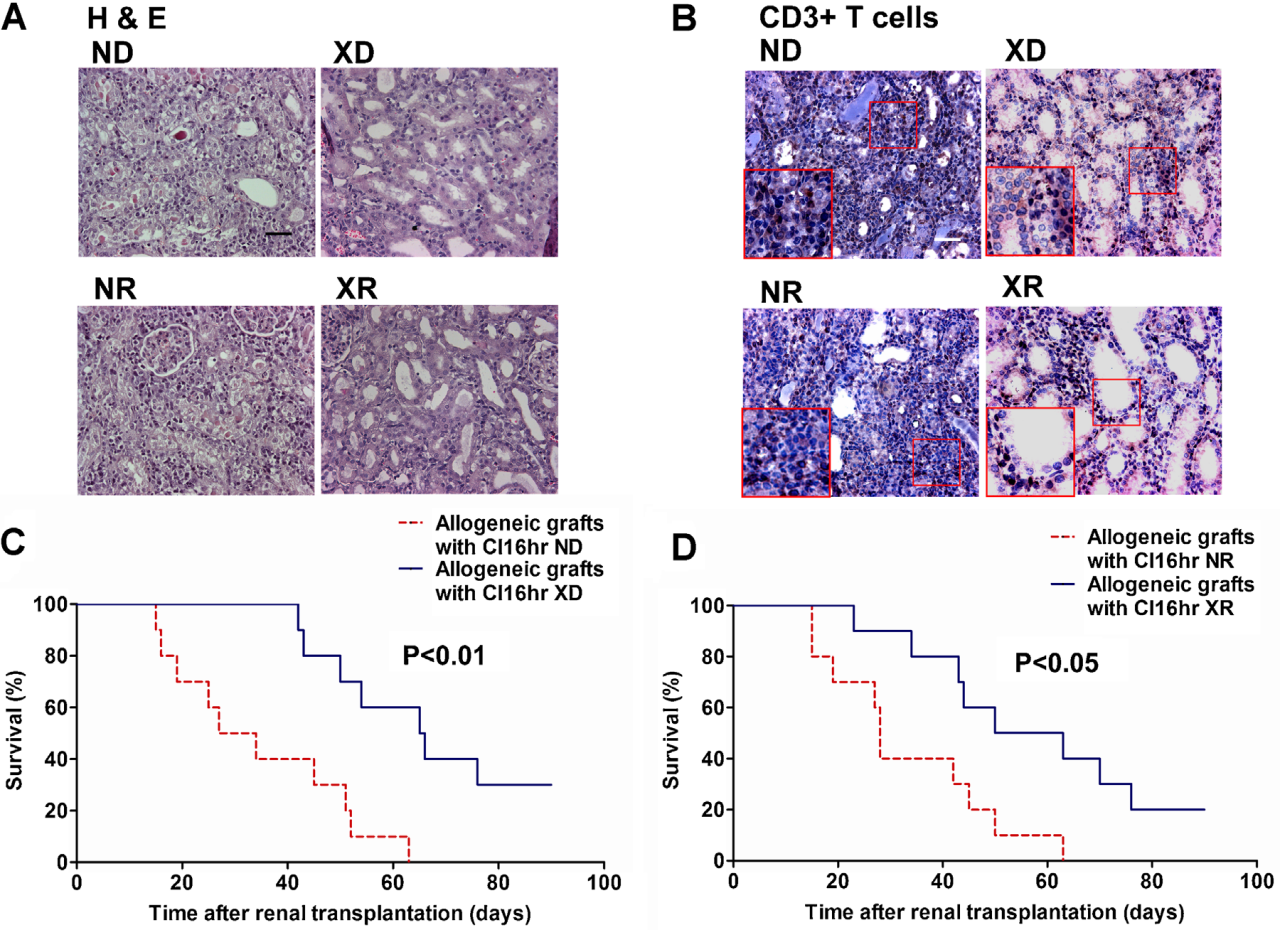
Xenon treatment prolonged the allogeneic grafts survival. Xenon treatment (70% xenon balanced with 30% oxygen) was given either to Fischer donor animals before organ retrieval (XD) or Lewis recipient immediately after engraftment (XR). Grafts were kept in 4°C Soltran preserving solution (cold ischemia; CI) for 16 h. Donor or recipient exposed to nitrogen (70% N_2_ balanced with 30% O_2_) served as treatment control. Contralateral kidney was removed 4 days after transplant surgery. Allograft recipient received cyclosporin A (5 mg/kg) for 10 days. (A) Histology of renal graft on Day 20 after transplant surgery. (B) CD3^+^T cells assessed by immunohistochemistry on Day 20 after transplantation. Kaplan–Meier survival analysis of recipient animals in Fischer-to-Lewis allografts. Xenon gas was given to donor Fischer animals (C) before organ retrieval or to recipient immediately (D) after engraftment (n = 10). Scale bar: 50 μm.

## Discussion

Our findings represent the first report on the renoprotective effects of xenon against cold ischemia associated delayed graft function in rodent transplant model. Xenon given either to graft donors (pretreatment) or to recipients (posttreatment) prolonged graft survival. The underlying mechanism is very likely to be due to sustained upregulation of HIF-1α and its down-stream products which led to protection from tubular cell injury induced by IRI and subsequently enhanced tubular cell survival. Ultimately, these effects in turn dampened the inflammatory response via inhibition of the HMGB1/TLR4 signaling pathways.

Previous studies have shown that inhibiting HIF degradation via hydroxylases protected kidneys from ischemia-reperfusion injury and enhanced long-term kidney graft survival 16–17. Our previous study would suggest that xenon treatment does not activate HIF-1 via the HIF-1α degradation pathway but activated the up-stream mTOR pathways to accelerate HIF-1α synthesis 14. Moreover, targeting HIF results in the broad spectrum of protective effects through the master switch of several sets of down-stream genes such as erythropoietin (EPO) 18, VEGF 19 and heme oxygenase-1(HO-1) 20. Thus, manipulation of the HIF-1 pathway could be a promising approach to improve kidney transplant outcome.

Shortly after transplantation, expression of both HMGB-1 21 and its receptor TLR-4 22 are greatly enhanced in grafts with long cold preservation time, indicating that the full development of graft ischemia-reperfusion was dependent upon signaling through the HMGB-1/TLR-4 pathway 23–24 which promotes production of pro-inflammatory cytokines (TNF-α, IL-6 and IL-1β) and inflammation. Involvement of the HMGB-1/TLR-4 pathway is supported by evidence that TLR-4 deficient (TLR4−/−) mice are spared from kidney IRI and that the administration of a neutralizing antibody to HMGB-1 either before or soon after ischemia-reperfusion conferred significant protection 24. Our data demonstrate that HIF-1α up-regulation could stabilize the nuclear and cytoskeletal structure of tubular cells in response to oxidative and inflammatory stress and that this cellular structural stabilization restricts the HMGB-1 within the nuclei. This is consistent with a previous report that inhibition of HMGB-1 cytoplamic translocation protects cells from further damage 25. Deactivation of TLR-4/NF-κB would prevent the formation of inflammatory milieu, reflected by decreased systemic release of proinflammatory cytokines, such as TNF-α, IL-6 and IL-1β, and the further exacerbation of renal injury would be attenuated as shown in our study.

Our data clearly demonstrate that xenon counteracted the IRI-associated deleterious consequences of renal functional disturbance, which prevented the occurrence of delayed graft function. The long-term transplant survival is prolonged in both syngeneic and allogeneic grafts. Despite a wealth of clinical and laboratory evidence showing that transplanted kidneys with prolonged cold ischemia time are more susceptible to long-term deterioration 26–27, the precise mechanism underlying this correlation is still unclear. A likely explanation is that the initial ischemia-reperfusion injury severely diminishes the functional nephron reserves, Increasing workload on the remaining nephrons would result in suboptimal efficiency of blood filtration and gradual accumulation of toxic metabolites and harmful oxidative byproducts within the cells 28. This renders nephrons more susceptible to injury and accelerates the cell aging, resulting in further reduction of functional renal mass 29. In addition, the vigorous cell death initiated by IRI causes the massive release of DAMPs such as HMGB-1, and induces heavy influx of infiltrating immunocompetent cells, such as neutrophils and macrophages. This in turn aggravates the injury to the tubular cells and produces more cell components readily presented by MHC molecules on dendritic cells and macrophages 30, accelerating antigen processing and presenting to T cells, which mount the alloresponse 31. As chronic reduction of nephron populations further progresses, the compensatory changes would eventually fail to preserve homeostasis, proteinuria could prevail until the graft fails. Based on this, we could conclude that xenon could terminate this self-perpetuating cycle of nephron ablation by enhancing cell survival and proliferation and constraining the release of DAMPs, which could potentially elevate the immunogeneity of the post-ischemic kidney and agitate adaptive immunity. Indeed, our data indicated that the xenon treatment attenuates the T cell response (Figure 10B), possibly through its cytoprotection, which in turn decreases graft immunogenicity. This warrants further study. The renal functional reserve could be raised and sufficient functioning nephrons could survive through the subsequent incoming immune and nonimmune insults.

Our study is not without limitations. First, the PI3K/Akt/HIF-1α pathways after engraftment were not explored in this study and hence the further impact of HIF1α siRNA on those pathways and xenon-induced protection during IRI is open to a question. This warrants further study. Second, HIF-1α up-regulation is not unique to xenon as such effect has been reported with other anesthetics, for example, isoflurane 32,33. However, the profound depressing effects of isoflurane on cardiovascular and respiratory system can be an obstacle for clinical use in vulnerable transplanted patients.

Xenon has been used as an anesthetic gas clinically for several decades and is known to have a remarkable safety profile 8. All these suggest that xenon is readily suitable for clinical trial in the kidney transplant setting. Although the high cost may be an obstacle for its wide use, this can be overcome by using a recycle system 35 and hence, the cost to benefit ratio is very likely to be decreased when compared to the high cost of dialysis. Arguably, our data reported here indicated that xenon pretreatment could provide the optimal protection in terms of timing and efficacy while posttreatment may be more feasible with cadaveric donor graft. If our data can be extrapolated to the clinical setting, then translation of this strategy into clinic may likely improve the long-term transplant perspectives through minimizing the early graft injury.

## Abbreviations

DAMPs: damage associated molecular pattern molecules
DGF: delayed graft function
HIF-1: hypoxia inducible factor-1
HMGB-1: high-mobility group box -1
IRI: ischemia-reperfusion injury
siRNA: small interfering RNA
TLR-4: toll-like receptor 4
VEGF: vascular endothelial growth factor.

## References

[b1] Meier-Kriesche HU, Schold JD, Srinivas TR, Kaplan B (2004). Lack of improvement in renal allograft survival despite a marked decrease in acute rejection rates over the most recent era. Am J Transplant.

[b2] Li C, Yang CW (2009). The pathogenesis and treatment of chronic allograft nephropathy. Nat Rev Nephrol.

[b3] Quiroga I, McShane P, Koo DD (2006). Major effects of delayed graft function and cold ischaemia time on renal allograft survival. Nephrol Dial Transplant.

[b4] Siedlecki A, Irish W, Brennan DC (2011). Delayed graft function in the kidney transplant. Am J Transplant.

[b5] Eltzschig HK, Eckle T (2011). Ischemia and reperfusion—From mechanism to translation. Nat Med.

[b6] Perico N, Cattaneo D, Sayegh MH, Remuzzi G (2004). Delayed graft function in kidney transplantation. Lancet.

[b7] Gluba A, Banach M, Hannam S, Mikhailidis DP, Sakowicz A, Rysz J (2010). The role of Toll-like receptors in renal diseases. Nat Rev Nephrol.

[b8] Lachmann B, Armbruster S, Schairer W (1990). Safety and efficacy of xenon in routine use as an inhalational anaesthetic. Lancet.

[b9] Hartlage MA, Berendes E, Van Aken H, Fobker M, Theisen M, Weber TP (2004). Xenon improves recovery from myocardial stunning in chronically instrumented dogs. Anesth Analg.

[b10] Preckel B, Mullenheim J, Moloschavij A, Thamer V, Schlack W (2000). Xenon administration during early reperfusion reduces infarct size after regional ischemia in the rabbit heart in vivo. Anesth Analg.

[b11] Ma D, Hossain M, Pettet GK (2006). Xenon preconditioning reduces brain damage from neonatal asphyxia in rats. J Cereb Blood Flow Metab.

[b12] Ma D, Hossain M, Chow A (2005). Xenon and hypothermia combine to provide neuroprotection from neonatal asphyxia. Ann Neurol.

[b13] Homi HM, Yokoo N, Ma D (2003). The neuroprotective effect of xenon administration during transient middle cerebral artery occlusion in mice. Anesthesiology.

[b14] Ma D, Lim T, Xu J (2009). Xenon preconditioning protects against renal ischemic-reperfusion injury via HIF-1alpha activation. J Am Soc Nephrol.

[b15] Li Q, Verma IM (2002). NF-kappaB regulation in the immune system. Nat Rev Immunol.

[b16] Hill P, Shukla D, Tran MG (2008). Inhibition of hypoxia inducible factor hydroxylases protects against renal ischemia-reperfusion injury. J Am Soc Nephrol.

[b17] Bernhardt WM, Gottmann U, Doyon F (2009). Donor treatment with a PHD-inhibitor activating HIFs prevents graft injury and prolongs survival in an allogenic kidney transplant model. Proc Natl Acad Sci USA.

[b18] Sharples EJ, Thiemermann C, Yaqoob MM (2005). Mechanisms of disease: Cell death in acute renal failure and emerging evidence for a protective role of erythropoietin. Nat Clin Pract Nephrol.

[b19] Pugh CW, Ratcliffe PJ (2003). Regulation of angiogenesis by hypoxia: Role of the HIF system. Nat Med.

[b20] Blydt-Hansen TD, Katori M, Lassman C (2003). Gene transfer-induced local heme oxygenase-1 overexpression protects rat kidney transplants from ischemia/reperfusion injury. J Am Soc Nephrol.

[b21] Ilmakunnas M, Tukiainen EM, Rouhiainen A (2008). High mobility group box 1 protein as a marker of hepatocellular injury in human liver transplantation. Liver Transpl.

[b22] Kruger B, Krick S, Dhillon N (2009). Donor Toll-like receptor 4 contributes to ischemia and reperfusion injury following human kidney transplantation. Proc Natl Acad Sci USA.

[b23] Wu H, Ma J, Wang P (2010). HMGB1 contributes to kidney ischemia reperfusion injury. J Am Soc Nephrol.

[b24] Wu H, Chen G, Wyburn KR (2007). TLR4 activation mediates kidney ischemia/reperfusion injury. J Clin Invest.

[b25] Yang H, Rivera Z, Jube S (2010). Programmed necrosis induced by asbestos in human mesothelial cells causes high-mobility group box 1 protein release and resultant inflammation. Proc Natl Acad Sci USA.

[b26] Salahudeen AK, Haider N, May W (2004). Cold ischemia and the reduced long-term survival of cadaveric renal allografts. Kidney Int.

[b27] van der Vliet JA, Warle MC, Cheung CL, Teerenstra S, Hoitsma AJ (2011). Influence of prolonged cold ischemia in renal transplantation. Clin Transplant.

[b28] Helal I, Fick-Brosnahan GM, Reed-Gitomer B, Schrier RW (2012). Glomerular hyperfiltration: Definitions, mechanisms and clinical implications. Nat Rev Nephrol.

[b29] Mackenzie HS, Tullius SG, Heemann UW (1994). Nephron supply is a major determinant of long-term renal allograft outcome in rats. J Clin Invest.

[b30] Burne-Taney MJ, Yokota N, Rabb H (2005). Persistent renal and extrarenal immune changes after severe ischemic injury. Kidney Int.

[b31] Lu CY, Penfield JG, Kielar ML, Vazquez MA, Jeyarajah DR (1999). Hypothesis: Is renal allograft rejection initiated by the response to injury sustained during the transplant process. Kidney Int.

[b32] Zhang L, Huang H, Cheng J (2011). Pre-treatment with isoflurane ameliorates renal ischemic-reperfusion injury in mice. Life Sci.

[b33] Hieber S, Huhn R, Hollmann MW, Weber NC, Preckel B (2009). Hypoxia-inducible factor 1 and related gene products in anaesthetic-induced preconditioning. Eur J Anaesthesiol.

[b34] Raphael J, Zuo Z, Abedat S, Beeri R, Gozal Y (2008). Isoflurane preconditioning decreases myocardial infarction in rabbits via up-regulation of hypoxia inducible factor 1 that is mediated by mammalian target of rapamycin. Anesthesiology.

[b35] Rawat S, Dingley J (2010). Closed-circuit xenon delivery using a standard anesthesia workstation. Anesth Analg.

